# Optimizing crop management strategies for improved yield, water productivity, and sustainability of quinoa in shallow basaltic semi-arid regions

**DOI:** 10.3389/fpls.2025.1522995

**Published:** 2025-03-10

**Authors:** Aliza Pradhan, Jagadish Rane, P. S. Basavaraj, Neeraj Kumar, Dhanashri Shid, Nobin C. Paul, K. K. Pal, K. Sammi Reddy

**Affiliations:** ^1^ School of Drought Stress Management, ICAR-National Institute of Abiotic Stress Management, Pune, Maharashtra, India; ^2^ ICAR-Central Institute for Arid Horticulture, Bikaner, Rajasthan, India

**Keywords:** quinoa, semi-arid, shallow basaltic, sustainability, water productivity, yield

## Abstract

**Introduction:**

Recently, quinoa (*Chenopodium quinoa* Willd.) has gained global recognition as a nutritious, adaptable crop suitable to adverse soil and climatic conditions. However, knowledge about optimal management practices for its cultivation in marginal areas of India is limited.

**Methods:**

In this context, a field experiment was conducted in a split-split plot design with four sowing dates (D_1_: 1st November; D_2_: 15th November; D_3_: 1st December, D_4_: 15th December) in main plots, two irrigation levels (I_1_: 40% ET_c_; I_2_: 80% ET_c_) in sub-plots, and three nitrogen doses (N_1_: 100 kg N ha^-1^; N_2_: 150 kg N ha^-1^; N_3_: 200 kg N ha^-1^) in sub-sub plots having three replications during 2021-22 and 2022-23 in shallow basaltic *murram* soils.

**Results and discussion:**

Results indicated that sowing on 1st November yielded the highest seed production of 1446 kg ha^-1^, as temperatures aligned closely with optimal growth conditions. Quinoa's drought tolerance meant that deficit irrigation was able to maintain the crop growth and yield. While the crop responded positively to higher N doses, the study found that applying 100 kg N ha^-1^ was optimal, considering shallow basaltic soil conditions and potential lodging issues. Additionally, water productivity, protein, and saponin content reflected similar trends to seed yield. The results suggested that early sowing, irrigation at 40% ET_c_, and 100 kg N ha^-1^ produced a seed yield of 1446 kg ha^-1^, demonstrating higher carbon efficiency and sustainability while minimizing N_2_O emissions. However, these strategies should be tailored to specific agro-ecological conditions. Overall, the findings confirm quinoa’s potential for cultivation in India’s 26 million hectares of shallow basaltic *murram* soils, where other crops may not thrive economically.

## 1 Introduction

Recently, global agriculture has been emphasized by adopting climate-resilient and environmentally sustainable practices while aiming to reduce low greenhouse gas and carbon emissions. However, the dilemma lies between feeding a growing population and depletion of its natural resource base, particularly in water-scarce environments of semi-arid and arid regions. In emerging economies experiencing population growth like India, there is an urgent need to address food and nutrition demands amid climate variability (Srivastava et al., 2022). In this context, encouraging climate-smart, nutritious crop production systems is crucial for providing accessible, affordable, safe, and nutritious diets for communities. In recent years, quinoa (*Chenopodium quinoa* Willd.) has been gaining global attention as a highly nutritious agro-industrial crop, capable of thriving in adverse soil and climatic conditions (Fuentes and Bhargava, 2011; Hinojosa et al., 2018; Langyan et al., 2023). Due to its stress tolerance mechanism and minimal water requirement (300–400 mm), the crop is considered highly suitable for marginal areas of arid and semi-arid regions (Bhargava et al., 2006). Compared to dominant cereals such as rice, wheat, and maize, quinoa stands out as a gluten-free pesudocereal ^1^ rich in protein (13%–17%), well-balanced amino acids, essential vitamins, minerals, and bioactive compounds (Sindhu and Khatkar, 2019). Although quinoa's outer seed coat contains the bitter toxic compound saponin (0.1%–5%), which must be removed before consumption, it has significant industrial value due to its diverse biological activities, including antifungal, antiviral, anticancer, hypocholesterolemic, hypoglycemic, antithrombotic, diuretic, and anti-inflammatory effects (Vilcacundo and Hernández-Ledesma, 2016). Quinoa's resilience and superior nutritional profile have positioned it as a promising crop to combat silent hunger and malnutrition while reducing the global food environmental footprint (FAO, 2011). Further, cultivation of the crop demands minimal investment, and its yield potential could enhance farmers' profitability and resilience in climate change-affected environments.

Since the United Nations' declaration of the International Year of Quinoa in 2013, there has been a rapid expansion in the cultivated area dedicated to this crop, shifting perceptions and elevating its status from a minor to a potentially major crop (Bazile et al., 2016). With the expansion of quinoa cultivation to over 120 countries, most of the scientific studies have focused on location-specific technological aspects of crop production. While quinoa is well-suited to a variety of agro-climatic conditions, identifying the optimal planting date is crucial for successful cultivation in a given region. Most studies have suggested that the sowing window from October to December is ideal, with November being the optimal planting month in arid and semi-arid regions (Ramesh et al., 2019; Maamri et al., 2022). However, a few studies have highlighted January (Asher et al., 2020), as well as March–May, as preferable sowing windows for the crop (Taaime et al., 2023). Additionally, the stabilization of quinoa yields through deficit irrigation has been emphasized in studies by Garcia et al. (2003), Geerts et al. (2008); Pathan et al. (2023), and Mirsafi et al. (2024). Regarding nitrogen fertilization, research indicates an optimal nitrogen rate ranging from 90 kg N ha^−1^ to 225 kg N ha^−1^, depending on cultivar, management practices, and soil and environmental conditions (Kaul et al., 2005; Salim et al., 2019; Berti et al., 2000; Wang et al., 2020; Keshtkar et al., 2022). However, a positive increase in seed yield with higher irrigation levels combined with increased nitrogen doses has been reported by Bahrami et al. (2022) and AbdElgalil et al (2023). Further, Bhargava et al. (2006) highlighted quinoa's potential for both agricultural and industrial applications, particularly in India. Given that a substantial portion of the Indian population lacks access to protein-rich diets, quinoa's proteinaceous seed could significantly contribute to addressing hunger. The study also emphasized exploring the commercial potential of the crop for product development and marketing. However, to date, there have been limited developments in terms of quinoa's adaptation in India, despite the country's arid and marginal environments.

Edaphic constraints, such as shallow (26.4 million ha) and low-fertility soils (49.7 million ha), particularly in water-scarce and drought-prone agro-ecologies of peninsular India (Minhas and Obi Reddy, 2017), highlight the need for alternative crop-based interventions. Hence, this study was conducted to explore quinoa production techniques focusing on optimizing the sowing date, irrigation, and nitrogen management to ensure successful quinoa production in water-scarce marginal environments. The specific objective of our study was to assess the impact of these crop management strategies on quinoa yield, water productivity, quality, and environmental sustainability in shallow basaltic semi-arid regions.

## 2 Materials and methods

### 2.1 Study site

**Figure 1:**
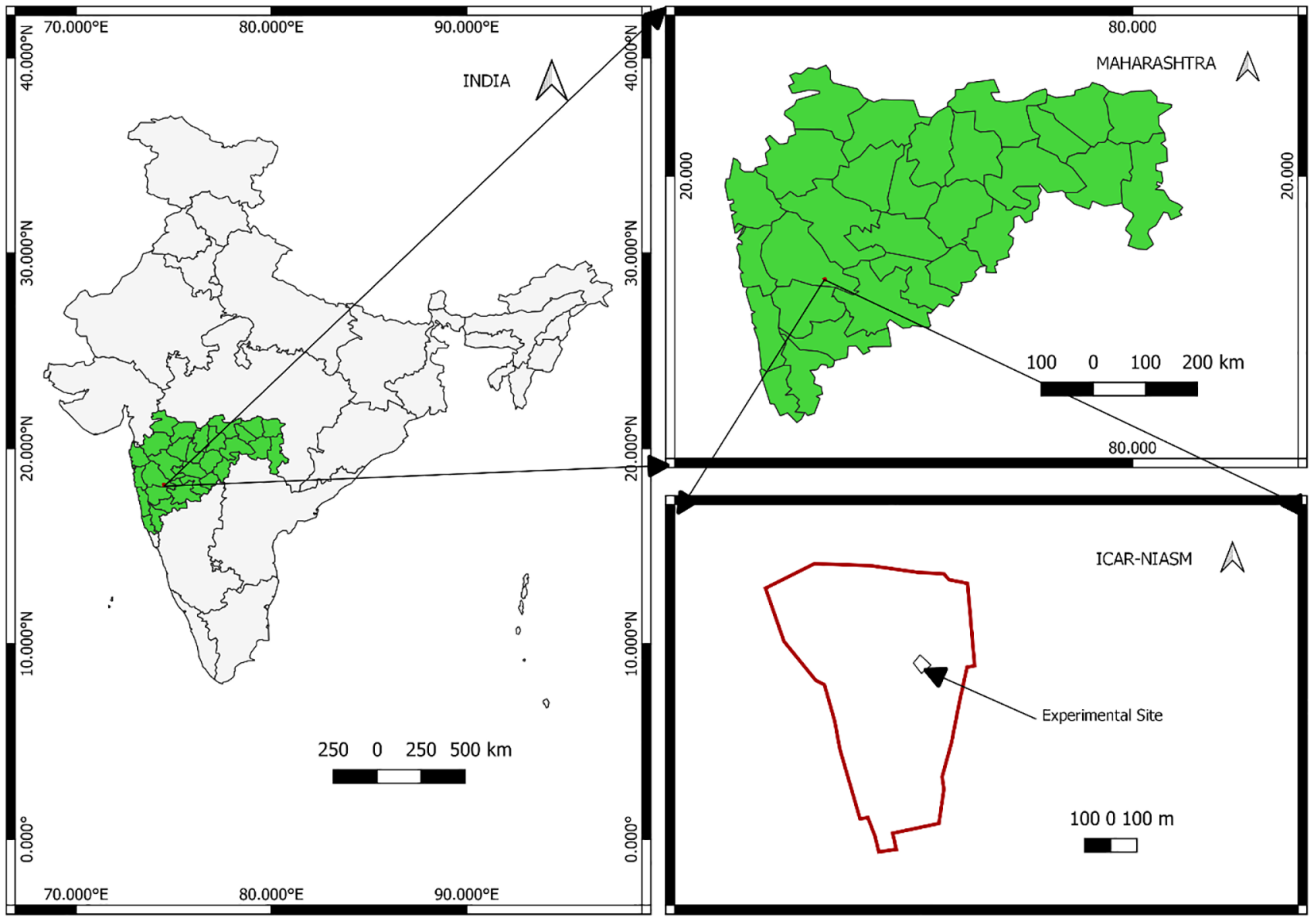
Location map of the study site.

The field experiment was carried out during 2021–2022 and 2022–2023 at ICAR-National Institute of Abiotic Stress Management (NIASM), India (18°09′30.62″ N latitude and 74°30′30.08″ E longitude, altitude of 570 m above mean sea level) (**Figure 1**). The site falls within the hot semi-arid agro-ecological region in the Deccan Plateau of India, known for its extremely high temperatures, unpredictable rainfall patterns, and extended periods of dry weather (Pradhan et al., 2023). Its long-term average annual rainfall is 576 mm, 70% of which occurs during June–September as southwest monsoons and 21% during October–December as retreating monsoons. The average values for temperature (maximum and minimum), relative humidity (maximum and minimum), and total rainfall and open pan evaporation during the crop growing periods (November–March) were 30.8°C and 15.7°C, 84% and 39%, and 8.2 and 23.0 mm during 2021–2022 and 31.5°C and 14.3°C, 79.9% and 30.4%, and 7.8 mm and 25.1 mm during 2022–2023, respectively (Automatic Weather Station, ICAR-NIASM). The details of the weather parameters are provided in **Supplementary Table 1**. The soil of the experimental site is originated from parental basaltic rocks and characterized as shallow *murrum* (up to 40-cm depth) with 30% stones (>2 mm): 69% sand, 21% silt, 10% clay, and 5% available moisture holding capacity. At the beginning of the experiment, the pH (1:2.5 soil:water), electrical conductivity (EC), Walkley–Black carbon (C), KMnO_4_ oxidizable nitrogen (N), 0.5 M NaHCO_3_ extractable phosphorous (P), and 1 N NH_4_OAc extractable potassium (K) were 7.2, 0.18 dS m^−1^, 0.14%, 98.32 kg ha^−1^, 2.51 kg ha^−1^, and 207 kg ha^−1^, respectively.

### 2.2 Experimental details and crop management

The experiment was laid out in a split-split plot design with four sowing dates (D_1_, November 1; D_2_, November 15; D_3_, December 1, D_4_, December 15) in main plots, two irrigation practices (I_1_, 40% ET_c_; I_2_, 80% ET_c_) in sub plots, and three nitrogen doses (N_1_, 100 kg N ha^−1^; N_2_, 150 kg N ha^−1^; N_3_, 200 kg N ha^−1^) in sub-sub plots having three replications (**Supplementary Figures 1**, **2**). The nitrogen quantity was applied in two splits: 50% as basal and 50% during the flowering period. The P and K doses (i.e., 60 kg ha^−1^) were applied as basal in all the treatments. The experiment was initiated in November 2021 using the quinoa accession "Jaipur Local", manually sown at a seed rate @ 5 kg ha^−1^ with 45 cm × 15 cm spacing. Sowing was established by dibbling five to six seeds per pocket in the soil to a depth of 1–2 cm. The gross and net plot areas under each treatment were 9 × 5 m and 7.5 × 4 m, respectively. To avoid border and interaction effects, a buffer area of 1.5 m was left between experimental units. Irrigation to the crops was provided via surface drip irrigation (having a discharge rate of 4 L h^−1^ through 16-mm laterals having inline emitters), and the crops were irrigated based on the actual crop evapotranspiration (ET_c_) approach. The crop ET_c_ was computed using the following equation (Equation 1):


(1) $${\text{ET}}_{\text{c}}(\text{mm})={\text{K}}_{\text{p}}\times {\text{K}}_{\text{c}}\times {\text{E}}_{\text{pan}}$$

where K_p_ is the pan coefficient (0.70), E_pan_ is the cumulative pan evaporation (mm), and K_c_ is the crop-specific coefficient. The K_c_ values for quinoa as reported by Garcia et al. (2003) were considered in our study. One common irrigation of 30 mm was provided after sowing for uniform germination and crop stand establishment. Scheduling of later irrigations was conducted as per the treatment, i.e., at 40% and 80% ET_c_ to quinoa. The total quantity of water applied through drip irrigation to the cropping systems in both the study years is given in **Supplementary Table 2**. All recommended crop-specific packages and practices of weeding, intercultural operations, and disease pest management were strictly followed.

When the seeds matured and plants started drying, all the morphological and yield attributing parameters were measured from a total of 10 plants per treatment. The shoots (only stem and leaves) and roots were oven-dried at 60°C for 48 h to determine the respective dry matter content. The crops were manually harvested using sickles as they matured, followed by sun drying for 7 days. The dried panicles for each experimental plot were threshed and winnowed manually. Seed yield was recorded at 12% moisture content, while the stover was oven-dried at 60°C until constant weight was obtained and then expressed as kg ha^−1^ for respective treatments.

### 2.3 Water productivity

Water productivity (WP) was calculated using the following equation (Equation 2).


(2) $$\text{WP}({\text{kg m}}^{-3})=\frac{\;\text{Economic yield}(\text{kg ha}-1)}{\;\text{Total water applied}(\text{m}3\;\text{ha}-1)\;}$$

### 2.4 Phenology and growing degree days

The phenological developments, *viz.*, 50% germination, 50% visible bud, 50% flowering, and 50% maturity were recorded as and when at least 50% of the plants were showing the indications in the whole plot. Days to maturity was calculated from the date of emergence to the date when the crop was harvested.

Growing degree days (GDD) was calculated based on the following formula (Equation 3):


(3) $$\text{GDD}=[({\text{T}}_{\text{max}}+{\text{T}}_{\text{min}})/2]-{\text{T}}_{\text{base}}$$

where T_max_ is the maximum temperature, T_min_ is the minimum temperature, and T_base_ is the base temperature, which was 3°C for quinoa (Jacobsen and Bach, 1998).

### 2.5 Protein and saponin contents in quinoa seed and husk

The seed protein content was determined using the Kjeldahl method of N estimation from plant samples with a conversion factor of 6.25. The seed protein content was then multiplied with the seed yield to estimate the protein yield under corresponding treatments. The total saponin content of quinoa seed and husk was estimated following the colorimetric determination procedure reported by Hiai et al. (1976). All quinoa samples were ground with a blender and passed through 1-mm sieve. Then, 10 g of each sample powder was dissolved in 40 mL of 25% ethanol and kept in a mechanical shaker for 12 hrs. Then, 0.5 mL of vanillin solution was added to 0.5 mL of aqueous ethanol sample, followed by the addition of 5 mL of 72% sulfuric acid and mixed in an ice-water bath. The mixture was then warmed in the bath at 60°C for 10 minutes, followed by cooling in an ice-water bath. A water blank with the reagents was also made. Absorbance at 450 nm was recorded against the blank with the reagents using a spectrophotometer. Quillaja saponin was used as a standard, and the total saponin content was expressed as g 100 g^−1^ of dry weight.

### 2.6 Carbon budgeting, efficiency, sustainable index, and nitrous oxide emissions

The total C input and output were determined by adding the carbon equivalents of all inputs and outputs during crop production. Carbon equivalents (CE) of all inputs, operational activities, and processes were combined to estimate the source-wise contribution to C input (**Supplementary Figure 3**; **Supplementary Table 3**). Similarly, the C output of each cropping system was quantified using methodologies provided by Choudhary et al. (2017) and Equation 4.


(4) $$\text{Carbon output}({\text{kg CE ha}}^{-1})=\text{Total biomass}(\text{seed}+\text{stover}){\text{yield in kg ha}}^{-1}\times 0.44$$

considering that C content accounts for 44% of the total plant biomass as given by Lal (2004).

Carbon efficiency, an indicator of total C production over the total input C, was calculated as follows (Equation 5).


(5) $$\text{Carbon efficiency}=\frac{\text{ Total C output}(\text{kg CE ha}-1)}{\text{Total C input}(\text{kg CE ha}-1)\;}$$

Carbon sustainability index (CSI) was estimated as the net gain in C over the total C input as given by Lal (2004) and Choudhary et al. (2017) and depicted in Equation 6.


(6) $$\text{CSI}=\frac{\;(\text{Total C output}-\text{Total C input})}{\text{Total C input}}$$

Carbon footprint (CF): Carbon footprints of the cropping systems indicated the total greenhouse gas (GHG) emissions in terms of kg CE ha^−1^ to produce 1 kg of the economic product and was computed as per Equation 7 as suggested by Yadav et al. (2021).


(7) $$\text{CF}=\frac{\text{Total carbon input}(\text{kg CE ha}-1)}{\text{Economic yield}(\text{kg ha}-1)\;}$$

Estimation of nitrous oxide (N_2_O) emission included both direct and indirect emissions. As direct N_2_O emission is proportional to the amount of N applied, direct N_2_O emission was computed using Equation 8.


(8) $${\text{Direct N}}_{2}\text{O}({\text{kg CO}}_{2}-{\text{eq ha}}^{-1})=\text{Quantity of N fertilizer}({\text{kg ha}}^{-1})\times 0.016\times 1.571\times 298$$

where 0.016 is the default emission factor for N fertilizer application, 1.571 is the conversion factor used to convert N_2_ to N_2_O, and 298 is the global warming potential (GWP) of N_2_O concerning CO_2_.

Similarly, indirect N_2_O emissions included loss of N fertilizer in the form of volatilization and were calculated using the Intergovernmental Panel on Climate Change (IPCC) guidelines of Tier 1 and Equation 9.


(9) $${\text{Indirect N}}_{2}\text{O}({\text{kg CO}}_{2}-{\text{eq ha}}^{-1})=\text{Quantity of N fertilizer}({\text{kg ha}}^{-1})\times 0.1\times 0.010\times 1.571\times 298$$

where 0.1 is the fraction used for volatilization, 0.010 is the default emission factor used for volatilization, 1.571 is the conversion factor used to convert N_2_ to N_2_O, and 298 is the GWP of N_2_O concerning CO_2_.

### 2.7 Statistical analysis

The recorded data were statistically analyzed using analysis of variance (ANOVA) for split-split plot design using the "Agricolae" package of R (R Development Core Team, 2015). A mixed model was used considering sowing date as the main plot factor, irrigation as sub plot factor, and nitrogen management as the sub-sub plot factor and analyzed in a split-split plot design. Since no major differences were observed among the treatments for the recorded observations, the results were averaged for 2021–2022 and 2022–2023. The F-test and least significant difference (LSD) (p < 0.05) were used to decipher the significance of the means of treatments and their interactions.

## 3 Results

### 3.1 Quinoa phenology

**Figure 2:**
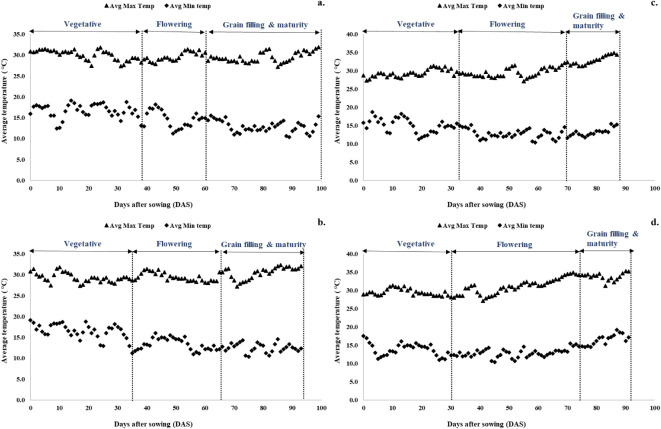
Effect of date of sowing on quinoa phenological stages. **(a)** November 1, **(b)** November 15, **(c)** December 1, and **(d)** December 15.

The impact of different sowing dates on quinoa's growth duration, developmental stages, and corresponding temperature ranges is illustrated in **Figure 2**. For sowing on November 1, the vegetative stage continued for 38 days after sowing (DAS), with temperatures ranging from a maximum of 27.35°C to 31.45°C and a minimum of 12.45°C to 19.1°C. The flowering period extended over 22 days, with temperatures reaching a maximum of 27.9°C to 31.4°C and a minimum of 11.25°C to 18.2°C. The seed-filling and maturity stages began at 60 DAS and continued for 33 days, resulting in a total crop duration of 99 days. During this phase, maximum temperatures ranged from 28°C to 31.5°C, while minimum temperatures ranged from 10.35°C to 14.9°C. Sowing on November 15 reduced the total crop duration by 1 week compared to November 1. The vegetative stage lasted 35 days, with a wider window for flowering (30 days). The seed-filling and maturity stages started at 65 DAS and lasted for 33 days. Temperatures during these stages were as follows: vegetative stage (27.35°C to 31.8°C maximum and 11.25°C to 19.1°C minimum), flowering stage (28°C to 31.4°C maximum and 11°C to 16.05°C minimum), and seed-filling and maturity stages (27.15°C to 32.1°C maximum and 10.35°C to 14.5°C minimum), respectively. Sowing in December resulted in a longer flowering period (35–40 days) and shorter seed-filling and maturity periods (20–23 days). During the seed-filling and maturity phases, temperatures were notably higher: 31.3°C to 34.85°C maximum and 11.6°C to 15.25°C minimum for December 1 sowing and 31.25°C to 35.25°C maximum and 14.5°C to 19.15°C minimum for December 15 sowing.

### 3.2 Cumulative growing degree days

For cumulative growing degree days (CGDD), the highest accumulation was observed for the earliest sowing date, November 1, with 1,900.40°C (**Table 1**). Sowing on November 15, December 1, and December 15 resulted in lower CGDD values of 1,785.30°C, 1,661.75°C, and 1,789.25°C, respectively.

**Table 1:** Cumulative growing degree days (CGDD in °C) of different phenological stages for different sowing dates in quinoa.

Quinoa phenological stages	November 1	November 15	December 1	December 15
	(CGDD in °C)			
50% germination	208.05	331.33	155.58	96.85
50% visible buds	868.28	698.46	670.31	595.25
50% flowering	1,021.73	925.41	936.19	1,031.38
50% maturity	1,486.01	1,377.26	1,339.44	1,434.38
Harvesting	1,900.40	1,785.30	1,661.75	1,789.25
Crop duration (days)	99	93	87	91

### 3.3 Plant height, dry matter accumulation, and yield attributes

Sowing date and nitrogen levels showed significant effects (p < 0.05) on quinoa height, dry matter accumulation, and yield attributes (**Table 2**). The maximum plant height (113.67 cm) was obtained during the November 1 sowing. Height decreased by 7%–20% with later sowing dates, reaching a minimum (91.28 cm) for sowing on December 15. A similar trend was seen for both shoot and root dry matter, ranging from 11.39 to 72.43 g plant^−1^ and 3.52 to 8.69 g plant^−1^, respectively. Among the yield attributes, sowing on November 1 produced the maximum panicle weight, panicle length, seed weight, and husk weight, which were 2.6, 1.7, 7.2, and 2.7 times, respectively, than those from December 15 sowing (19.05 g plant^−1^, 18.24 cm plant^−1^, 10.15 g plant^−1^, and 4.70 g plant^−1^). However, irrigation levels did not significantly affect these parameters except panicle length, which was 8.8% higher in 80% ET_c_ than in 40% ET_c_ (22.93 cm plant^−1^) (**Table 2**). Among the nitrogen doses, the highest mean values for plant height, shoot dry matter, and yield attributes were achieved with N_3_ (200 kg N ha^−1^) and the lowest with N_1_ (100 kg N ha^−1^). The application of 150 kg N ha^−1^ was significant as compared to 100 kg N ha^−1^ only for shoot dry matter and seed weight. The maximum seed weight was observed under N_3_, which was 33% higher than that under N_2_ (39.25 g plant^−1^) and 56% higher than that under N_1_ (33.40 g plant^−1^). The seed weight of N_2_ was 18% higher than that of N_1_ (p < 0.05). Husk weight was significantly higher for November sowing (12. 8 g plant^−1^) and for 200 kg N ha^−1^ (11.95 g plant^−1^) (p < 0.05). The 1000-seed weight of quinoa ranged from 2.22 to 2.74 g, with plots sown in November recording a 17% higher value than that of December sown plots (2.2 g) (p < 0.05). Similarly, providing irrigation at 80% ET_c_ and 200 kg N ha^−1^ reported 10% and 7% higher 1000-seed weight than 40% ET_c_ (2.35 g) and 100 kg N ha^−1^ (2.39 g), respectively.

**Table 2:** Effect of sowing date, irrigation, and nitrogen levels on quinoa plant height, dry matter accumulation, and yield attributes.

Treatments	Plant height (cm)	Shoot dry matter (g plant^−1^)	Root dry matter (g plant^−1^)	Panicle weight (g plant^−1^)	Panicle length (cm plant^−1^)	Seed weight (g plant^−1^)	Husk weight (g plant^−1^)	1000-seed weight (g)
Date of sowing (D)								
D_1_: November 1	113.6 a	72.4 a	8.7 a	49.8 a	30.9 a	73.1 a	12.8 a	2.7 a
D_2_: November 15	104.8 b	64.5 a	7.9 a	33.3 b	23.7 b	46.4 b	12.6 a	2.6 a
D_3_: December 1	98.0 c	50.3 b	7.2 a	26.6 c	22.9 b	32.8 b	9.4 ab	2.3 b
D_4_: December 15	91.3 d	11.3 c	3.5 b	19.1 d	18.2 c	10.2 c	4.7 b	2.2 b
LSD (p < 0.05)	5.38	11.17	1.59	6.05	3.49	13.71	5.01	0.19
Irrigation levels (I)								
I_1_: 40% ET_c_	102.1 a	46.8 a	6.3 a	21.3 a	22.9 b	39.7 a	9.3 a	2.4 b
I_2_: 80% ET_c_	106.8 a	52.6 a	7.3 a	12.3 a	24.9 a	41.5 a	10.4 a	2.6 a
LSD (p < 0.05)	NS	NS	NS	NS	1.89	NS	NS	0.10
Nitrogen levels (N)								
N_1_: 100 kg ha^−1^	99.5 b	36.2 c	6.0 b	26.6 b	23.3 b	33.4 c	8.1 b	2.4 b
N_2_: 150 kg ha^−1^	103.7 ab	47.4 b	6.0 b	30.6 b	23.7 ab	39.3 b	9.5 b	2.4 b
N_3_: 200 kg ha^−1^	104.2 a	65.4 a	8.6 a	39.3 a	24.8 a	52.2 a	12.0 a	2.6 a
LSD (p < 0.05)	4.52	4.41	1.46	5.42	1.37	7.39	2.27	0.12

Means followed by different lowercase letters within a column are significantly different at p < 0.05 according to LSD test.

LSD, least significant difference.

### 3.4 Quinoa seed yield and water productivity

**Figure 3:**
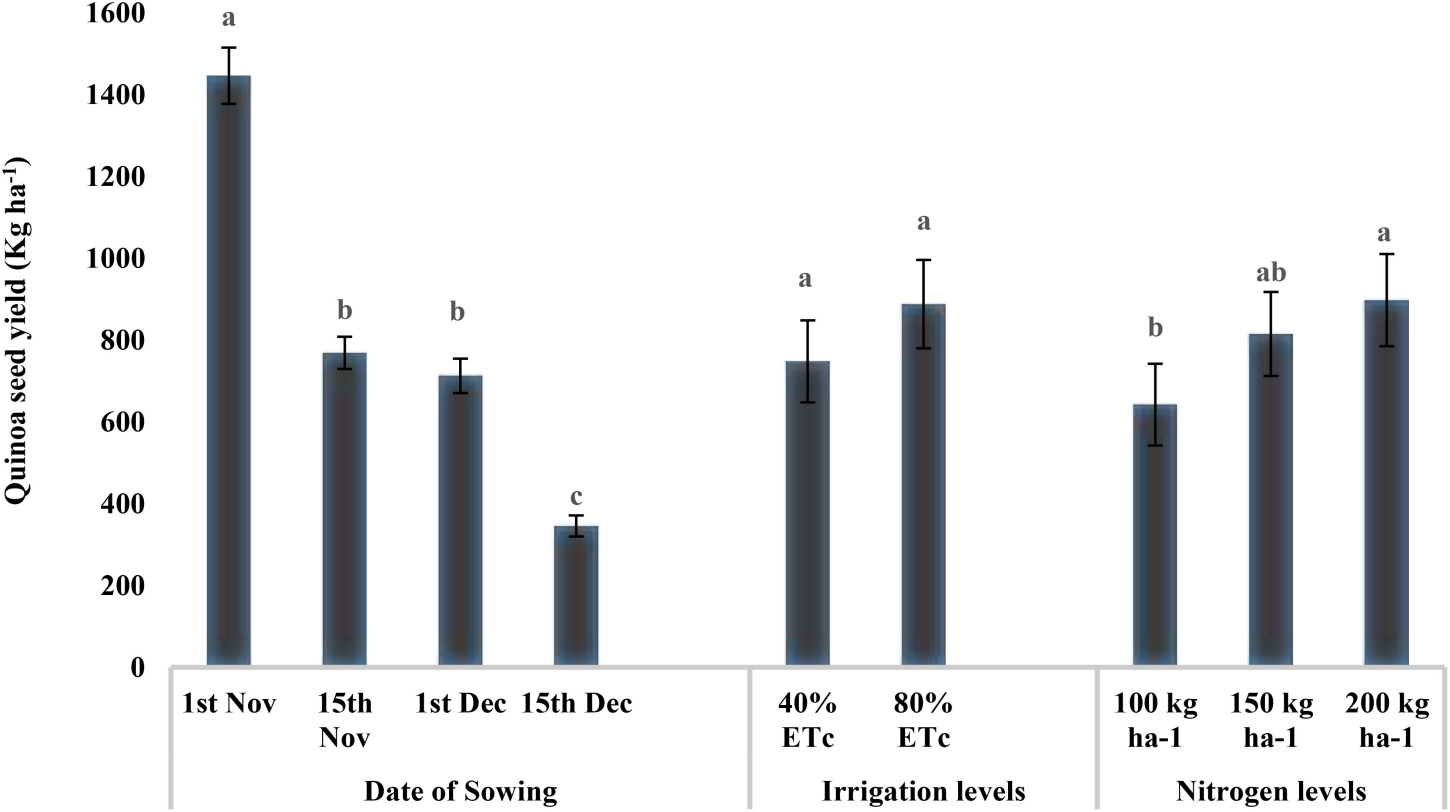
Effect of date of sowing, irrigation, and nitrogen levels on quinoa seed yield (kg ha^−1^). Vertical bars represent mean ± SE of the observed values. Values followed by different lowercase letters are significantly different at p < 0.05 within the treatment levels according to LSD test. LSD, least significant difference.

The seed yield was significantly higher in plots sown on November 1 (1,446 kg ha^−1^), which was reduced by 50% for sowings on November 15 and December 1 sowing, and the lowest yield was recorded for December 15 sowing (345.61 kg ha^−1^) (p < 0.05) (**Figure 3**). There were no differences in seed yield between the two irrigation levels. However, nitrogen levels had a significant effect on quinoa seed yield (p < 0.05) with maximum values under N_3_ (916.58 kg ha^−1^) and N_2_ (815.04 kg ha^−1^), with the latter being at par with N_1_ (723.50 kg ha^−1^).

**Figure 4:**
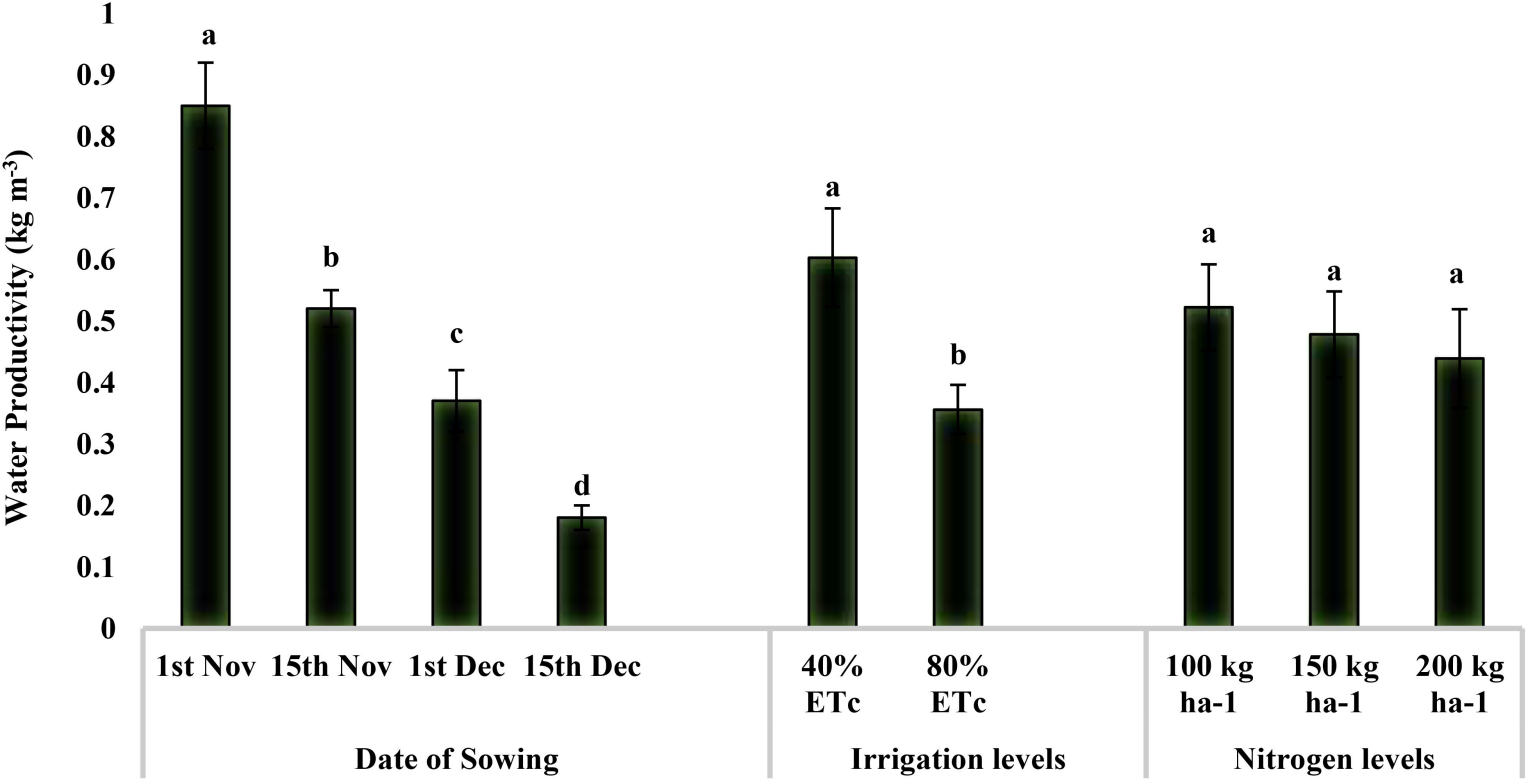
Effect of date of sowing, irrigation, and nitrogen levels on quinoa water productivity (kg m^−3^). Vertical bars represent mean ± SE of the observed values. Values followed by different lowercase letters are significantly different at p < 0.05 within the treatment levels according to LSD test. LSD, least significant difference.

The water productivity of quinoa production ranged from 0.85 kg m^−3^ to 0.18 kg m^−3^ for our study (**Figure 4**). Water productivity was maximum for November 1 sowing (0.85 kg m^−3^), followed by November 15 (0.52 kg m^−3^) and December 1 (0.37 kg m^−3^), and minimum for December 15 (0.18 kg m^−3^). Providing irrigation at 40% ET_c_ was 69.38% higher water productive than that at 80% ET_c_ (0.36 kg m^−3^). However, water productivity was not significantly influenced by the nitrogen levels.

### 3.5 Quinoa protein and saponin content

**Figure 5:**
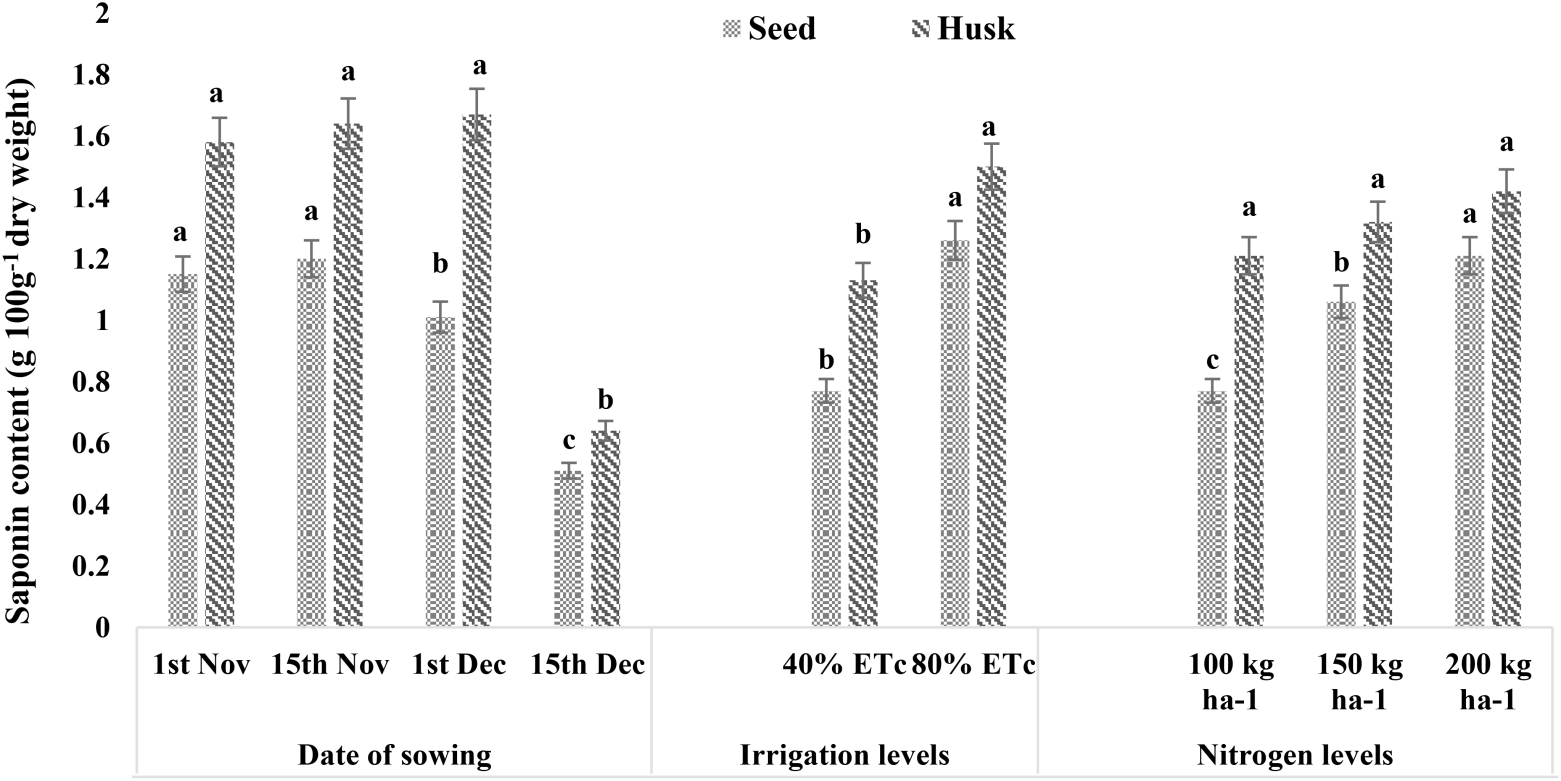
Effect of date of sowing, irrigation, and nitrogen levels on saponin contents of quinoa seed and husk (g 100 g^−1^ dry weight). Vertical bars represent mean ± SE of the observed values. Values followed by different lowercase letters are significantly different at p < 0.05 within the treatment levels according to LSD test. LSD, least significant difference.

Since the treatment had no significant effect on seed protein content, the system protein yield followed a similar trend as that of seed yield (**Supplementary Table 4**; **Supplementray Figure 4**). Seed and husk saponin contents evaluated in the current study ranged from 0.51 to 1.26 g per 100-g dry weight and 0.64 to 1.67 g per 100-g dry weight, respectively (**Figure 5**). The seed saponin content was 19% higher in November sown crops than in the December 1 sowing (1.01 g 100 g^−1^ dry weight). However, the minimum saponin content of 0.51 g 100 g^−1^ dry weight was reported for the December 15 sowing. Similarly, providing irrigation at 40% ET_c_ had 39% lower saponin as compared to that under 80% ET_c_ (1.26 g 100 g^−1^ dry weight). The application of a higher dose of N increased the seed saponin content with N_1_ having the lowest value (0.77 g 100 g^−1^ dry weight), followed by N_2_ (1.06 g 100 g^−1^ dry weight) and N_3_ (1.21 g 100 g^−1^ dry weight). The saponin content of husk was higher than that of seed with irrigation levels having a significant impact, following a similar trend to that of seed saponin content. Delayed sowing, i.e., on December 15, also reported a reduced husk saponin content than the rest of the sowing dates.

### 3.6 Carbon budgeting, efficiency, and sustainability index

Averaged over 2 years, the carbon budgeting and related indices differed among the treatments (**Table 3**). Early sowing dates, i.e., November 1 sowing (D_1_), had the lowest C footprint (0.19 kg CE kg^−1^ seed) and the highest C efficiency (3.69) and were more sustainable (CSI, 2.69) (p < 0.05). This may be due to improved biomass production leading to higher C output (1,018.26 kg CE ha^−1^) and moderate C input (276 kg CE ha^−1^) in S_1_. Sowing during December 15 (D_4_) resulted in the highest C footprint (0.80 kg CE kg^−1^ seed) and the lowest C efficiency (0.88) and was not sustainable (CSI, −0.12), which was due to significantly less C output (243.31 kg CE ha^−1^) as proportionate to the quantity of C input (279.53 kg CE ha^−1^). Similarly, irrigating the crop at 40% ET_c_ proved to be more C efficient and sustainable as compared to irrigating them at 80% Etc. Among the nitrogen levels, the application of 100 kg N ha^−1^ (N_1_) registered the lowest C footprint (0.33), higher C efficiency (2.14), and CSI (1.14), which was comparable to N_2_, i.e., 150 kg N ha^−1^. However, higher N levels, i.e., application at 200 kg ha^−1^ (N_3_), resulted in higher C output (632.07 kg CE ha^−1^) but at the cost of efficiency and increased C footprint; therefore, they were less sustainable (CSI, 0.86).

**Table 3:** Effect of date of sowing, irrigation, and nitrogen levels on carbon input–output parameters.

Treatments	Total C output (kg CE ha^−1^)	Total C input (kg CE ha^−1^)	Carbon footprint (kg CE kg^−1^ seed)	Carbon efficiency	Carbon sustainability index (CSI)
Date of sowing (D)					
D_1_: November 1	1,018.2 ^a^	276.0 ^a^	0.2 ^c^	3.7 ^a^	2.7 ^a^
D_2_: November 15	541.3^b^	274.0^a^	0.4^b^	1.9^b^	0.9^b^
D_3_: December 1	501.7^b^	276.2^a^	0.4^b^	1.9^c^	0.9^c^
D_4_: December 15	243.3^c^	279.5^a^	0.8^a^	0.9^d^	−0.1^d^
LSD (p < 0.05)	48.70	NS	0.05	0.03	0.06
Irrigation levels (I)					
I_1_: 40% ET_c_	526.6^a^	254.7^b^	0.3^b^	2.2^a^	1.2^a^
I_2_: 80% ET_c_	625.2^a^	286.7^a^	0.4^a^	1.9^b^	0.9^b^
LSD (p < 0.05)	NS	15.70	0.01	0.01	0.01
Nitrogen levels (N)					
N_1_: 100 kg N ha^−1^	452.1^b^	210.9^c^	0.3^b^	2.1^a^	1.1^a^
N_2_: 150 kg N ha^−1^	573.8^a^	275.5^b^	0.3^b^	2.1^ab^	1.1^ab^
N_3_: 200 kg N ha^−1^	632.1^a^	340.6^a^	0.4^a^	1.9^b^	0.9^b^
LSD (p < 0.05)	67.32	21.50	0.02	0.03	0.01

Means followed by different lowercase letters within a column are significantly different at p < 0.05 according to LSD test.

LSD, least significant difference.

### 3.7 Nitrous oxide emissions

**Figure 6:**
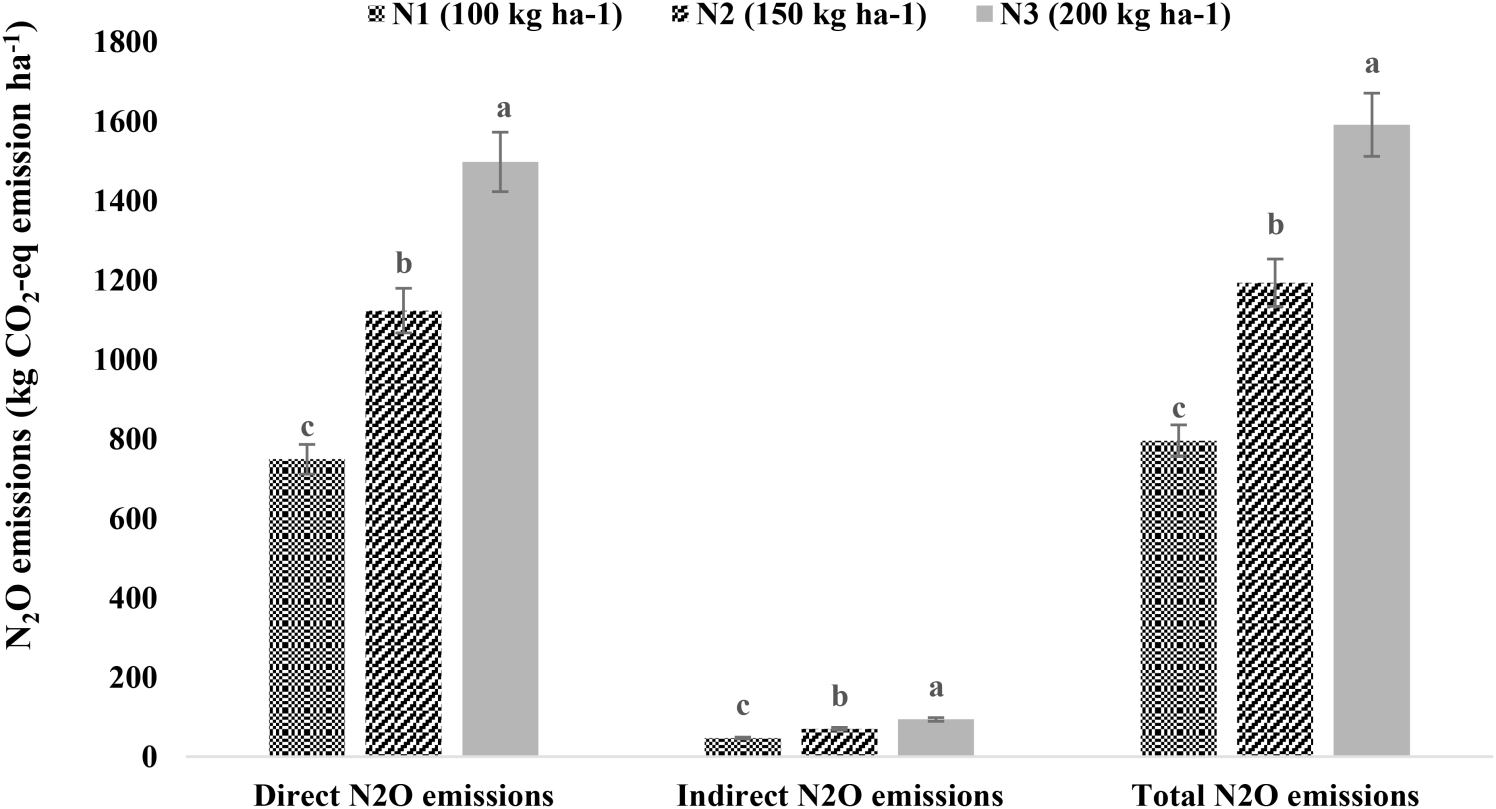
Nitrous oxide (N_2_O) emissions as affected by nitrogen levels. Means followed by different lowercase letters are significantly different at p < 0.05 according to LSD test. LSD, least significant difference.

Among the treatments, N_2_O emissions (both direct and indirect) were significantly influenced only by N levels (**Figure 6**). The N_2_O emissions increased with the applied N fertilizer dose. The application of 200 kg N ha^−1^ had maximum direct (1,497.15 kg CO_2_-eq ha^−1^), indirect (93.57 kg CO_2_-eq ha^−1^), and total (1,590.72 kg CO_2_-eq ha^−1^) N_2_O emissions, followed by 150 and 100 kg N ha^−1^ (p < 0.05).

## 4 Discussions

Optimizing production technology is essential for achieving the highest economic returns from any crop introduced to a new agro-ecological region. In this study, we evaluated how various sowing dates, irrigation regimes, and nitrogen management strategies affected quinoa yield, water productivity, and quality in shallow basaltic regions with a semi-arid climate. Our findings indicated significant differences in these parameters under different management scenarios. Although quinoa has the potential to tolerate a wide range of temperatures (−8°C to 35°C), this tolerance varies depending on the genotype and developmental stages. A sudden increase in temperature during the critical stages of the crop, i.e., flowering and seed filling, can significantly reduce yield and poses a major limitation to quinoa's global expansion (Dao et al., 2020). High temperatures at anthesis are crucial for quinoa pollination and can reduce pollen production and viability (Jacobsen et al., 2003). Temperatures above 35°C leading to substantial yield reductions due to empty seeds and seeds lacking inflorescence, reabsorption of quinoa seed endosperm, and inhibition of anther dehiscence has been reported by Bonifacio (1995). Even temperatures above 30°C hinder quinoa growth and productivity by reducing photosynthetic activity, flowering rates, and seed filling, leading to lower yields, as has already been highlighted by Hirich et al (2014). In our study, the average temperatures during critical growth stages for November 1 sowing were close to quinoa's optimal growth range (20°C–25°C), which may have led to better phenological development, growth, and yield. These findings are in agreement with Choukr-Allah et al. (2016) and Alvar-Beltrán et al. (2019). For early sowing dates (November 1 and 15), the maximum temperatures recorded were approximately 30°C during the anthesis, seed-filling, and maturity stages. However, December sowing experienced higher temperatures (34°C–35°C) during these stages, resulting in decreased yield and water productivity due to a shortened life cycle, with earlier flowering and improper seed maturation (Maestro-Gaitán et al., 2022; Matías et al., 2021). The temperature variations observed during this time of year and at this location were typical of tropical semi-arid zones. The length of the growing period varied with sowing dates, with the longest period occurring when sown on November 1 (99 days) and the shortest when sown on December 1 (87 days). This growing period was shorter compared to that observed in subtropical regions, which had durations of 169 and 134 days (Hassan, 2015; Präger et al., 2018). Regarding CGDD, the values reported in our study were comparable to those for similar agro-climatic regions (Präger et al., 2018). A higher accumulation of degree days among early sown plants of quinoa was also reported by Alvar-Beltrán et al. (2019).

In terms of irrigation, there was no significant difference in seed yield between 80% and 40% of crop evapotranspiration (ET_c_), as quinoa, being drought-tolerant, can thrive with limited water availability. By limiting water applications, this practice aims to enhance the water productivity and stabilize yields rather than maximize them (Geerts and Raes, 2009) and has been well investigated as an important and sustainable practice for arid and semi-arid regions (Garcia et al., 2003; Geerts et al., 2008). In contrast, other reports indicate that deficit irrigation can reduce seed yield by up to 50% compared to full irrigation (Hirich et al., 2012, Hirich et al., 2013). The variable response of irrigation on quinoa seed yield may be attributed to genotypes, soil, climate, and other crop management practices. However, crop WP ranged from 0.18 to 0.85 kg m^−3^ and was 67% higher under 40% ET_c_ compared to 80% ET_c_ (0.36 kg m^−3^). These findings align with the results reported by Fghire et al. (2013), confirming quinoa's high water use efficiency under drought-stress conditions. Quinoa's physiological responses to drought include rapid stomatal closure, sunken stomata, restricted root growth, and accelerated leaf senescence, which contribute to its adaptability in dry environments (Jacobsen et al., 2003).

Nitrogen is a well-known key factor influencing total plant biomass. However, optimizing crop yields with increased nitrogen rates depends on factors such as soil type, location, and management practices. In our study, significant differences were observed in various crop morphological traits, yield attributes, and seed yield between nitrogen rates of 100 kg ha^−1^ and 200 kg ha^−1^. However, both these doses were comparable to the moderate dose of 150 kg ha^−1^. Since the seed yield at 100 kg N ha^−1^ was at par with that of 150 kg N ha^−1^, indicating no proportional yield increase with an additional 50 kg N ha^−1^ application, 100 kg N ha^−1^ was considered optimal for our study. No differences in crop water productivity were found at higher nitrogen applications (150 and 200 kg ha^−1^) likely due to lower yield gain in proportion to the amount of water applied. Further, crop lodging (personal observation) occurred in plots receiving 200 kg N ha^−1^, likely due to increased plant biomass and the shallow soil depth at the study site, which restricted root growth and hindered proper anchorage. Similar reports of crop lodging with higher doses of N application were also reported by Wang et al. (2022). Our findings also align with reports from Kaul et al. (2005) and Shams (2012), which indicate that while quinoa yields and biomass increase with higher nitrogen application, they stabilize at a specific dosage for a given agro-ecological condition. In semi-arid regions, where water and nitrogen are crucial limiting factors, maintaining a well-developed crop canopy under full irrigation with high nitrogen doses is not sustainable. Therefore, leveraging the combined benefits of limited soil fertility and deficit irrigation can create a more effective strategy. Thus, for shallow basaltic regions using deficit irrigation, recommending a nitrogen application rate of 100 kg ha^−1^ will have optimum economic yield.

The 1000-seed weight observed in this study (2.22–2.74 g) is comparable with findings from other field studies (Tan and Temel, 2018) but lower compared to ranges reported for the Andean regions (3.0 g–4.7 g) (Miranda et al., 2012), which may be due to difference in terms of genotypes and pedo-climatic conditions. In general, early sowing is conducive to better seed filling and seed weight compared to late sowing. Therefore, the lower seed weight under late sowing dates can be attributed to the shortened seed-filling phase, where increased temperatures and longer photoperiods likely played significant roles. Other studies have also reported reduced 1000-seed weight due to limited irrigation and lower nitrogen application (Hirich et al., 2014b; Shams, 2018). Further, the seed and husk saponin content in our study falls within the range typically reported for quinoa (Pulvento et al., 2012; De Santis et al., 2016). The decrease in seed saponin content with delayed sowing may be related to the length of the crop growing period. Short-duration quinoa genotypes with lower seed saponin content (0.62 g 100 g^−1^ DM) were found to have less saponin compared to long-duration genotypes with higher content (1.92 g 100 g^−1^ DM), as noted by Oustani et al. (2023). Studies also reported that quinoa under water-deficit conditions tends to have lower saponin content, indicating better quality (Soliz-Guerrero et al., 2002; Gómez et al., 2011), which aligns with our results on irrigation levels. Further, saponin content with a positive and significant relationship with N dose has already been reported by Bilalis et al. (2012) and González et al. (2020).

Considering the impact of climate change and human-induced greenhouse gas emissions, promoting crop management practices that are more efficient and sustainable with minimal carbon footprints is essential (Yadav et al., 2021). The carbon input–output parameters reported in this study revealed that the early date of sowing, irrigation at 40% ET_c_, and the application of N @ 100 kg ha^−1^ were more C efficient and sustainable. This may be attributed to lesser emissions from irrigation and nitrogen coupled with higher proportionate C output. Similarly, the N_2_O emissions (both direct and indirect) increased in proportion to nitrogen fertilizer application. A significant and positive correlation of N_2_O emissions with N fertilizer application under drip irrigation was also reported by Kumar et al. (2021). Therefore, implementing optimal water and nutrient management strategies could stabilize N_2_O emissions while enhancing the carbon footprint and efficiency of quinoa production in shallow basaltic regions.

## 5 Conclusion

In shallow basaltic semi-arid regions, sowing quinoa on November 1, i.e., when temperatures align more closely with optimal quinoa growth conditions, can enhance crop biomass, yield, and water productivity. Higher temperatures during critical growth stages, i.e., anthesis and seed filling, and a short growing cycle are among the factors that reduced quinoa's yield in late November and December sowing. Therefore, planning agricultural activities, particularly through a well-planned sowing calendar, is crucial for quinoa cultivation so that temperatures during the critical growth stages must be as close as possible to the mean optimal temperatures. In our study, quinoa's growth, development, and yield were unaffected by irrigation levels. Therefore, frequent irrigation, but in small quantities, is highly suggested to reduce evapotranspiration and increase water productivity in quinoa in shallow basaltic *murram* soils. Further, nitrogen application at 100 g N ha^−1^ was found suitable considering the shallow basaltic rock, root restrictions, limited irrigation, and lodging issues. These optimization strategies are location-specific and can be tailored according to particular agro-ecological situations. However, the results confirm quinoa's ability to produce seed yields up to 1,446 kg ha^−1^, a level of production that most food crops cannot achieve economically in the shallow basaltic rocky terrains of drought-prone environments. This makes quinoa a promising candidate for crop diversification in India and other countries with similar climatic conditions. Furthermore, there is also a need to design a product marketing strategy and raise awareness among farmers and government agencies about quinoa's potential as a stress-tolerant alternative crop for marginal environments.

## Data Availability

The original contributions presented in the study are included in the article/**Supplementary Material**. Further inquiries can be directed to the corresponding author.

## Footnotes

1 The quinoa plant produces starchy seeds that resemble grains and are prepared and consumed in a similar manner, although botanically, it is classified as a dicotyledon.
