# Supplementary material for: Optimizing crop management strategies for improved yield, water productivity, and sustainability of quinoa in shallow basaltic semi-arid regions
Source: Front Plant Sci. 2025 Mar 10;16:1522995. doi: 10.3389/fpls.2025.1522995 (PMC11931028; doi:10.3389/fpls.2025.1522995)
Supplement: Supplementary file 1 [file DataSheet1.docx]

**Supplementary Tables and Figures**

**Supplementary Table 1**

Monthly meteorological parameters recorded during crop growing period of 2021-22 and 2022-23

| Crop growing period | 2021-22 | | | | | | 2022-23 | | | | | |
| --- | --- | --- | --- | --- | --- | --- | --- | --- | --- | --- | --- | --- |
|  | T_max_ | T_min_ | RH_max_ | RH_min_ | Total Rainfall | OPE^*^ | T_max_ | T_min_ | RH_max_ | RH_min_ | Total Rainfall | OPE |
|  | (°C) | (°C) | (%) | (%) | (mm) | (mm) | (°C) | (°C) | (%) | (%) | (mm) | (mm) |
| November | 30.4 | 18.8 | 83.7 | 52.7 | 2.8 | 4.1 | 30.5 | 14.8 | 81.8 | 34.1 | 0.0 | 4.6 |
| December | 28.2 | 14.8 | 91.6 | 51.0 | 5.0 | 2.7 | 30.4 | 15.2 | 85.0 | 39.6 | 1.4 | 3.8 |
| January | 27.8 | 12.8 | 89.2 | 43.1 | 0.0 | 3.6 | 30.3 | 13.0 | 85.7 | 33.7 | 0.4 | 4.3 |
| February | 31.9 | 13.5 | 82.0 | 28.0 | 0.0 | 5.6 | 32.9 | 12.8 | 74.0 | 21.1 | 0.0 | 5.8 |
| March | 35.9 | 18.7 | 73.6 | 20.4 | 0.4 | 7.0 | 33.3 | 15.4 | 73.0 | 23.3 | 6.0 | 6.6 |
| Mean/Total | 30.8 | 15.7 | 84.0 | 39.0 | 8.2 | 23.0 | 31.5 | 14.3 | 79.9 | 30.4 | 7.8 | 25.1 |

^*^OPE indicates USWB (United States Weather Bureau) Open Pan Evaporation

**
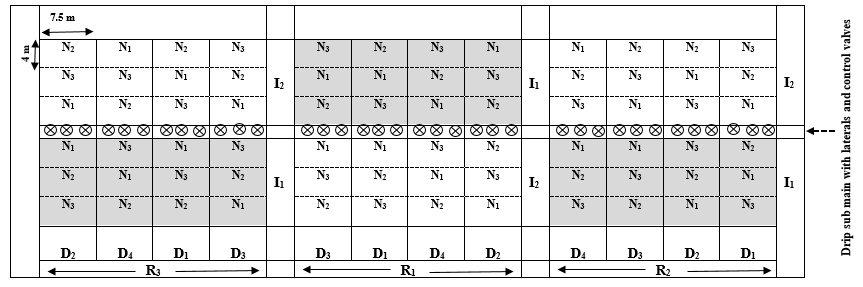
**

**Supplementary Figure 1**

Field layout of the experiment (D_1_: 1^st^ November; D_2_: 15^th^ November; D_3_: 1^st^ December, D_4_: 15^th^ December) in main plots, two irrigation practices (I_1_: 40% ET_c_; I_2_: 80% ET_c_) in sub plots, and three nitrogen doses (N_1_: 100 kg N ha^-1^; N_2_: 150 kg N ha^-1^; N_3_: 200 kg N ha^-1^) in sub-sub plots with three replications (R_1_, R_2_ and R_3_).


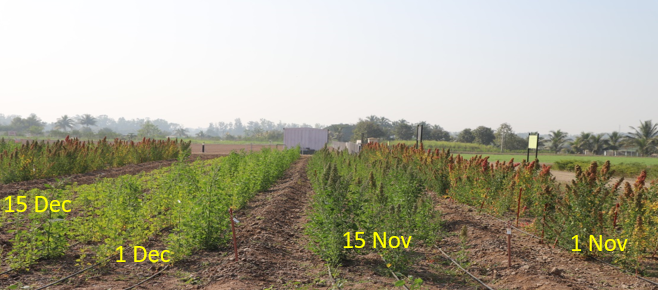


**Supplementary Figure 2**

Field view of the experiment

**Supplementary Table 2**

Effect of date of sowing on amount of water applied through irrigation (average for two years)

| **Date of sowing** | **Amount of irrigation water applied (mm)** | |
| --- | --- | --- |
|  | 40% ETc | 80% ETc |
| 1^st^ November | 109 | 218 |
| 15^th^ November | 125 | 250 |
| 1^st^ December | 133 | 266 |
| 15^th^ December | 140 | 280 |

**Supplementary Figure 3**

**Source-wise contribution to carbon input (%) in response to date of sowing (D), irrigation (I) and nitrogen levels (N) (Average data of two cropping seasons)** (D_1_: 1^st^ November; D_2_: 15^th^ November; D_3_: 1^st^ December, D_4_: 15^th^ December, I_1_: 40% ET_c_, I_2_: 80% ET_c_, N_1_: 100 kg N ha^-1^, N_2_: 150 kg N ha^-1^, N_3_: 200 kg N ha^-1^).

**Supplementary Table 3**

Carbon emission factors of agricultural inputs used in the study

| Sl No. | Particulars | Units | Equivalent carbon emission (kg CE unit^-1^) | References |
| --- | --- | --- | --- | --- |
| 1. | Farm machinery | h | 0.89 | Lal et al., 2020 |
| 2. | Diesel | L | 0.94 | Lal et al., 2020 |
| 3. | Irrigation water | m^3^ | 0.17 | Lal, 2004 |
| 4. | Seed | kg | 0.32 | Wang et al., 2015 |
| 5. | Human labour | Man day (8 h) | 0.23 | Lal et al., 2020 |
| 6. | Fertilizers |  |  |  |
| a. | Nitrogen (N) | kg | 1.30 | Lal, 2004 |
| b. | Phosphorous (P_2_O_5_) | kg | 0.20 | Lal, 2004 |
| c. | Potassium (K_2_O) | kg | 0.15 | Lal, 2004 |
| 7. | Plant Protection chemicals (fungicides) | Kg a.i. | 3.9 | Lal, 2004 |

**Supplementary Table 4**

Effect of date of sowing, irrigation and nitrogen levels on quinoa seed protein content (%).

| Treatments | Seed protein content (%) |
| --- | --- |
| Date of sowing (D) | |
| D_1_:1^st^ November | 15.7 |
| D_2_:15^th^ November | 15.2 |
| D_3_:1^st^ December | 15.3 |
| D_4_:15^th^ December | 15.4 |
| LSD (p < 0.05) | NS |
| Irrigation levels (I) | |
| I_1_:40% ETc | 15.5. |
| I_2_:80% ETc | 15.2 |
| LSD (p < 0.05) | NS |
| Nitrogen levels (N) | |
| N_1_:100 Kg ha^-1^ | 15.9 |
| N_2_:150 Kg ha^-1^ | 16.3 |
| N_3_:200 Kg ha^-1^ | 16.8 |
| LSD (p < 0.05) | NS |

**Supplementary Figure 4**

Effect of date of sowing, irrigation and nitrogen levels on protein yield (kg ha^-1^). Vertical bars represent mean ± SE of the observed values. Values followed by different lowercase letter are significantly different at p < 0.05 within the treatment levels according to LSD test.

**References**

Lal, B., Gautam, P., Panda, B.B., Tripathi, R., Shahid, M., Bihari, P., Guru, P.K., Singh, T., Meena, R.L., Nayak, A.K., 2020. Identification of energy and carbon efficient cropping system for ecological sustainability of rice fallow. Ecol. Indic. 115, 106431. doi: 10.1016/j.ecolind.2020.106431.

Lal, R., 2004. Carbon emission from farm operations. Enviorn. Int. 30(7), 981-990. Doi: 10.1016/j.envint.2004.03.005.

Wang, H., Yang, Y., Zhang, X., Tian G., 2015. Carbon Footprint Analysis for Mechanization of Maize Production Based on Life Cycle Assessment: A Case Study in Jilin Province, China. Sustainability. 7, 15772-15784.
